# The Impact of Elevated Atmospheric Carbon Dioxide Exposure on Magic Tomatoes’ Nutrition–Health Properties

**DOI:** 10.3390/ijms241612815

**Published:** 2023-08-15

**Authors:** Linda Boufeldja, Frederic Boudard, Karine Portet, Caroline Guzman, Sylvie Morel, Nathalie Berger, Orianne Duchamp, Claudie Dhuique-Mayer, Christian Dubos, Patrick Poucheret

**Affiliations:** 1 Qualisud, Université de Montpellier, Avignon Université, CIRAD, Institut Agro, IRD, Université de La Réunion, 34093 Montpellier, France; boufeldjalinda@gmail.com (L.B.); frederic.boudard@umontpellier.fr (F.B.); karine.portet@umontpellier.fr (K.P.); caroline.guzman@umontpellier.fr (C.G.); orianne.duchamp@gmail.com (O.D.); claudie.dhuique-mayer@cirad.fr (C.D.-M.); 2 Laboratoire de Botanique, Phytochimie et Mycologie, CEFE, CNRS-Université de Montpellier-Université Paul-Valéry Montpellier-EPHE-IRD, 34093 Montpellier, France; sylvie.morel@umontpellier.fr; 3 IPSiM, Université de Montpellier, CNRS, INRAE, Institut Agro, 34080 Montpellier, France; nathalie.berger@inrae.fr (N.B.); christian.dubos@inrae.fr (C.D.)

**Keywords:** tomato, magic, STUPICE, PLOVID, LA0147, nutrition, health, elevated atmospheric carbon dioxide

## Abstract

The release of carbon dioxide (CO_2_) into the atmosphere has accelerated during the last two decades. Elevated atmospheric CO_2_ concentration (eCO_2_) is known as an agent that improves plant photosynthesis. However, eCO_2_ was also correlated with alterations in the macronutrient and micronutrient compositions of various dietary crops. In order to explore the effect of eCO_2_ on the nutritional and health properties of tomatoes, three parental lines of the Magic population, which includes a large part of the genetic diversity present in large fruit varieties, were used as models. The plants were grown in growth chambers under ambient (400 ppm) or eCO_2_ (900 ppm) conditions. The macronutrient and micronutrient contents were measured. The anti-oxidant and anti-inflammatory bioactivities were assessed in vitro on activated macrophages. These analyses highlighted that the carbohydrate content was not affected by the eCO_2_, whereas the protein, carotenoid, lycopene, and mineral contents decreased. Regarding the anti-oxidant properties, no influence of eCO_2_ exposure was observed. Similarly, the anti-inflammatory properties were not affected by the eCO_2_. These data are in contrast with previous studies conducted on different plant species or accessions, indicating that the effect of eCO_2_ on crops’ nutrition and health properties is based on complex mechanisms in which growth conditions and genetic backgrounds play a central role.

## 1. Introduction

Currently, human activities are directly and indirectly threatening the global ecosystem and natural balance [1]. Increased population growth and fossil fuel consumption have accelerated carbon dioxide (CO_2_) production in the last two decades. Elevated CO_2_ (eCO_2_) has a remarkable impact on plant yield, as expressed by the increased biomass of C3 plants [2,3]. Atmospheric CO_2_ enrichment is known to be a fertilizing agent [4] that improves photosynthesis, especially in C3 plants. This effect is based on the modulation of key enzymes: the higher carboxylation rate of Rubisco and the repression of Ribulose-1,5-bisphosphate (RubP) [5]. Consequently, the biomass recorded in C3 plants may be twice as high as in other plants [6]. Furthermore, eCO_2_ was correlated with alterations in plant physiology, composition, and resistance, as well as decreases in nitrogen, phosphorus, and several trace elements [7]. Among the most noticeable changes at the macronutrient level, the plant composition under eCO_2_ exposure was characterized by a drop in protein content and a significant increase in carbohydrates [8]. The mechanism behind these alterations is not fully elucidated. Among the various hypotheses, a dilution effect and the inhibition of the transpiration process are proposed [8,9].

Such modifications may have an impact on agricultural productivity, plant quality, and food security. A higher sugar/protein ratio in eCO_2_ plants might be one of the primary drivers of nutrient imbalances in the human diet.

The high-sugar diets causing such alterations may have long-term health consequences on the population. An increased sugar intake may lead to the overconsumption of caloric intake and carbohydrate–homeostasis imbalance. It would contribute to the worsening of various pathophysiological conditions and metabolic disorders, such as insulin resistance, diabetes mellitus, metabolic syndrome, and associated comorbidities, e.g., cardiovascular diseases and cancers [10]. Similarly, a low-protein diet can negatively influence human health since proteins are involved in the homeostasis of bodily functions and structures. In addition, the high energy value of proteins reduces short-term food intake [11]. Several studies confirmed that a diet with an appropriate level of protein is recommended for weight loss and to reduce the risk factors for cardiovascular disease [12]. Zinc, nitrogen, and iron are considered key substances for many enzymatic reactions, modeling antioxidant defense and promoting organism balance [13]. A deficiency in these compounds could have a negative impact on biological homeostasis. Carotenoids and, in particular, lycopene, are known to be important metabolites with high free radical scavenging ability. Their protective effect against cardiovascular diseases and prostate cancer is well demonstrated [14]. Several studies have shown rising amounts of lycopene and β carotene in response to eCO_2_, while others have shown the opposite. Hence, the consequences of atmospheric CO_2_ enrichment for this type of pigment need further investigation [15].

All the previously demonstrated effects of eCO_2_ on crops’ quality and composition affect not only plants’ nutrient profiles, but also consumer health and bodily functions, such as redox status modulation and inflammatory responses. The changes in antioxidant activity induced by eCO_2_ were confirmed by several observations. Increases or decreases in this activity may vary as a function of the investigation method and of the molecules extracted [16]. Research about the impact of eCO_2_ on food plants and, consequently, on human health, is scarce. Few studies explored the influence of the level of plant exposure to CO_2_ on the potential modulation of these vegetal antioxidant, anti-inflammatory, and immunomodulatory bioactivities [17].

The tomato (*Solanum lycopersicum* L.) is the second-most heavily consumed crop in the world. Tomato fruits are known to be significant antioxidant food crops, with high contents of lycopene, carotenoids, vitamins, and phenolic compounds [18]. They are also known for their antifungal and anticarcinogenic properties [19,20], as well as for modulating lipotoxicity disorders and associated inflammatory processes [21].

In the present study, the effect of elevated CO_2_ concentrations on food quality was explored in several tomato food crops. We investigated the impact of eCO_2_ exposure on three tomato accessions, i.e., STUPICE, PLOVID, and LA0147, to explore the influence of elevated atmospheric carbon dioxide on their phytochemistry and bioactivity potential in response to this climate change parameter. These accessions were chosen because they represent a large part of the genetic diversity that is present in large fruit varieties. These genotypes are three parent germplasm of the “multiparent advanced generation intercross” (Magic) tomato population, which was designed for precise quantitative trait loci (QTL) mapping for geneticists and breeders [22,23].

## 2. Results

### 2.1. Mineral Contents

**Figure 1:**
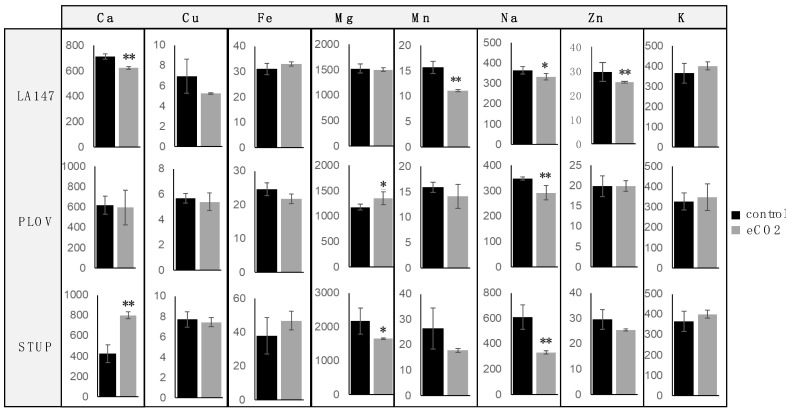
Minerals content of Magic tomato fruits (μg/g dry weight). Fruit samples were collected from 3 plants for each accession, and 30 fruits per plant grown under ambient 400 ppm of elevated 900 ppm atmospheric CO_2_ were used for analysis. One-way ANOVA followed by post hoc Tukey test or *t*-tests (significance: *, *p* < 0.05 and **, *p* < 0.01) were used.

The dosage of the mineral content in Magic tomato is presented in Figure 1. Tomato fruit concentration in eight mineral elements was measured: calcium (Ca), copper (Cu), iron (Fe), magnesium (Mg), manganese (Mn), sodium (Na), zinc (Zn), and potassium (K). In LA147 accession, a significant decrease in Ca, Mn, Na, and Zn was associated with eCO_2_ exposure. In PLOV accession, eCO_2_ exposure decreased Na but increased Mg. Regarding STUP accession, eCO_2_ exposure induced a decrease in Na and Mg content while Ca was increased. The other minerals in each accession were not statistically influenced by eCO_2_ exposure.

### 2.2. Micronutrients: Total Carotenoids and Lycopene Contents

**Figure 2:**
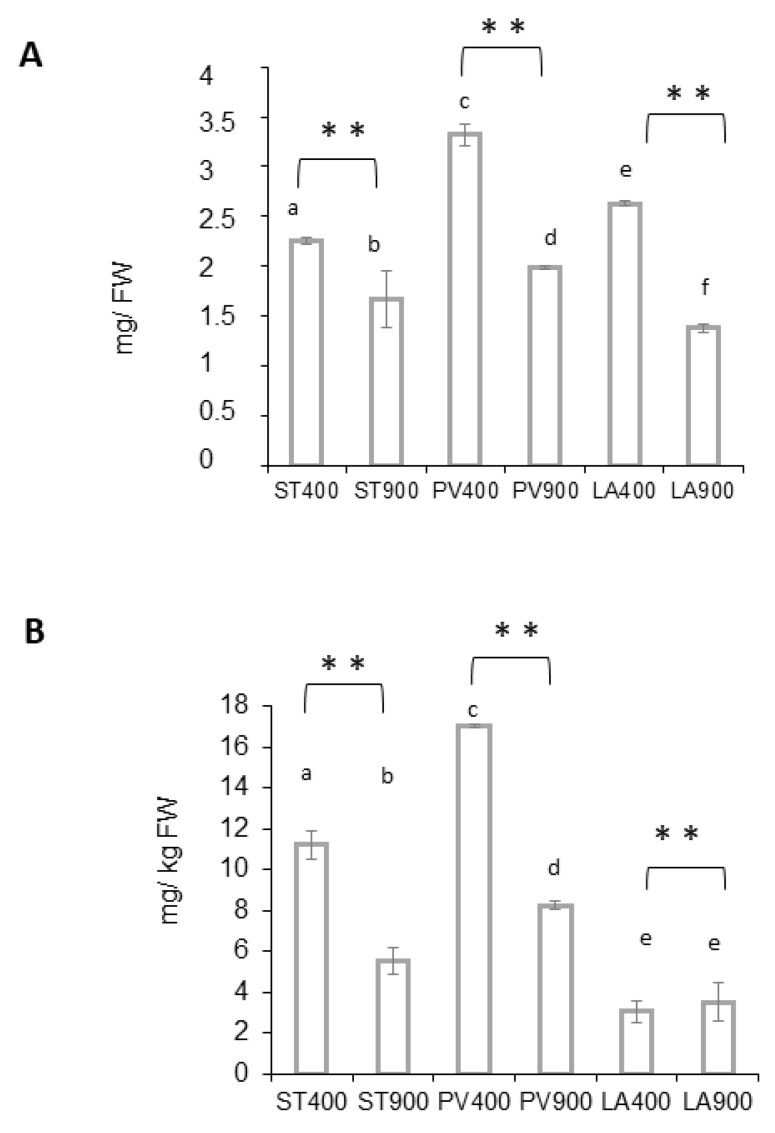
Carotenoids content of Magic tomato fruits. Fruit samples were collected from plants grown under ambient 400 ppm of elevated 900 ppm atmospheric CO_2_. (**A**) Total carotenoid content. (**B**) Lycopene content. Means within each condition with the same letter are not significantly different according to one-way ANOVA followed by post hoc Tukey test or *t*-tests (significance: **, *p* < 0.01).

The dosage of the Magic content in carotenoids is presented in Figure 2A. Significant differences in total carotenoids were recorded between the three Magic accessions. PLOVID presented the highest carotenoid content, followed by LA0147 and STUPICE. Magic samples grown under eCO_2_ contained significantly lower carotenoids when compared with ambient CO_2_ conditions. Lycopene (Figure 2B) measures confirmed that PLOVID and STUPICE pigment contents were superior to LA0147 under ambient CO_2_. While the latter was not affected by eCO_2_ exposure, the PLOVID and STUPICE samples showed a significant decrease in their lycopene content.

### 2.3. Macronutrients

#### 2.3.1. Sugars Content

**Figure 3:**
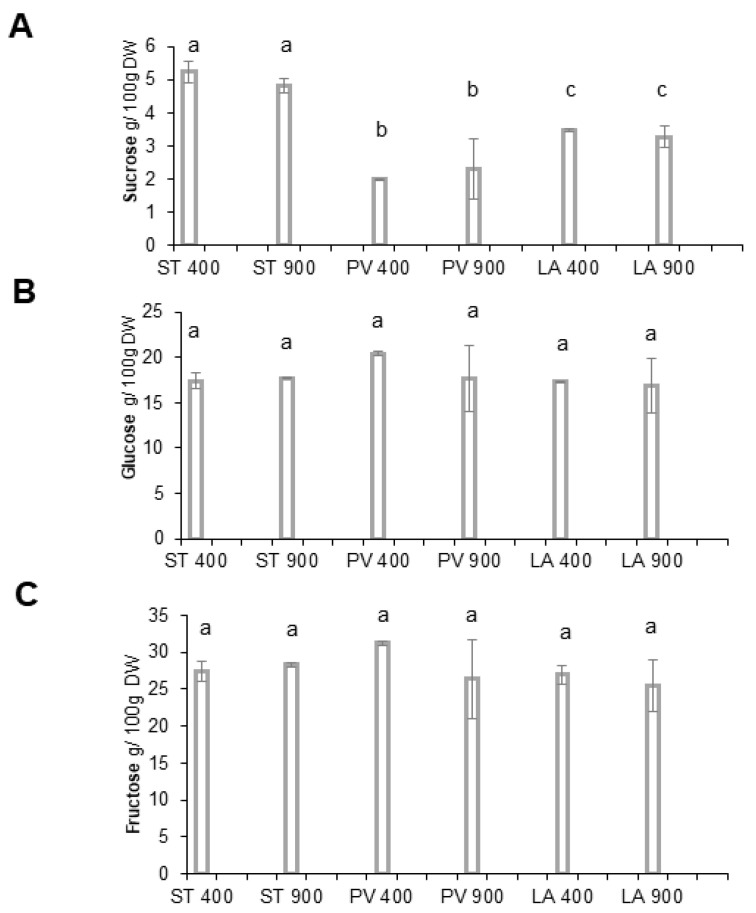
Carbohydrate content of Magic tomato fruits. Fruit samples were collected from plants grown under ambient 400 ppm of elevated 900 ppm atmospheric CO_2_. (**A**) Sucrose content; (**B**) glucose; and (**C**) fructose content. Means within each condition with the same letter are not significantly different according to one-way ANOVA followed by post hoc Tukey test or *t*-tests.

The results’ sugar measures (sucrose, glucose, and fructose) are presented in Figure 3. Regarding sucrose (Figure 3A), our data showed a significant difference in the sucrose content between the three accessions. The higher range of sucrose was identified in STUPICE. PLOVID is the second-richest, while LA0147 presented the lowest amount. The difference between the control CO_2_ samples and the eCO_2_ samples was not significant in all three accessions. The results for glucose (Figure 3B) indicate that the three Magic accessions presented the same content.

No significant difference between the two CO_2_ exposures was recorded between the three varieties. Fructose, shown in Figure 3C, was similar to glucose. eCO_2_ did not seem to have any significant impact on fructose accumulation in the three studied accessions. The fructose content remained unchanged between 21 and 31% in the three tomato samples.

#### 2.3.2. Total Lipid and Protein Content

**Figure 4:**
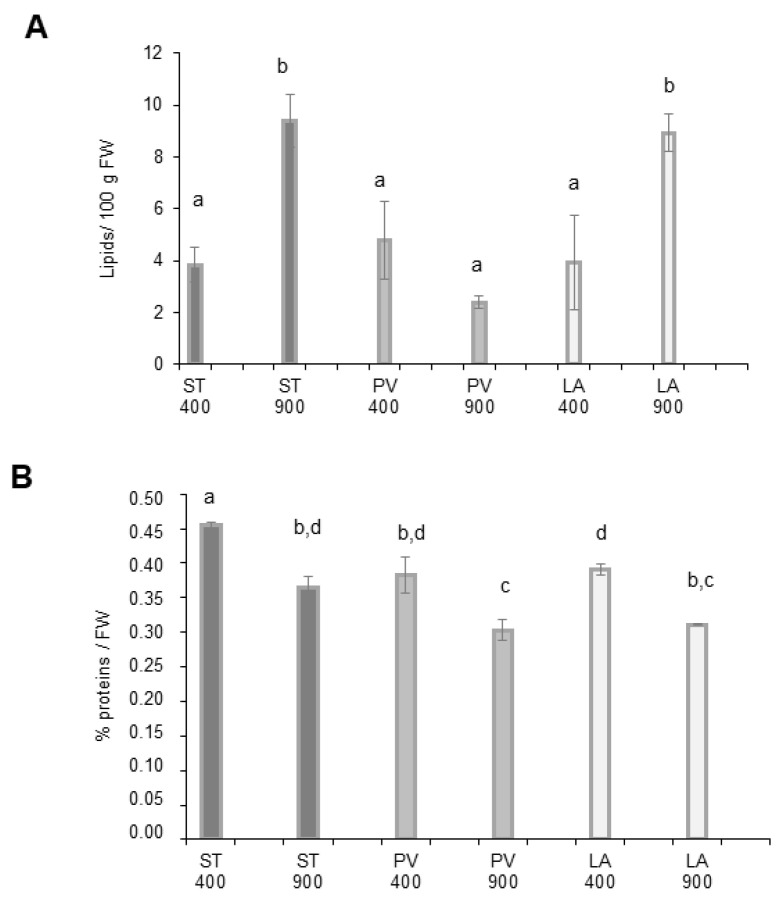
Lipid and protein contents of Magic tomato fruits. Fruit samples were collected from plants grown under ambient 400 ppm of elevated 900 ppm atmospheric CO_2_. (**A**) Total lipids. (**B**) Protein contents. Means within each condition with the same letter are not significantly different according to one-way ANOVA followed by post hoc Tukey test or *t*-tests (significance: *p* < 0.05).

Figure 4A shows the lipid measurement results. Magic varieties grown under ambient CO_2_ seemed to present the same quantity of total lipids. The total lipid remained unchanged after eCO_2_ exposure for the PLOVID variety. Conversely, the lipid content was significantly increased for both STUPICE and LA0147 upon eCO_2_ exposure. Protein content data are reported in Figure 4B. The results indicate that plants growing under eCO_2_ conditions present a significantly lower protein content compared with control conditions for the three Magic accessions.

### 2.4. Antioxidant Bioactivity

**Figure 5:**
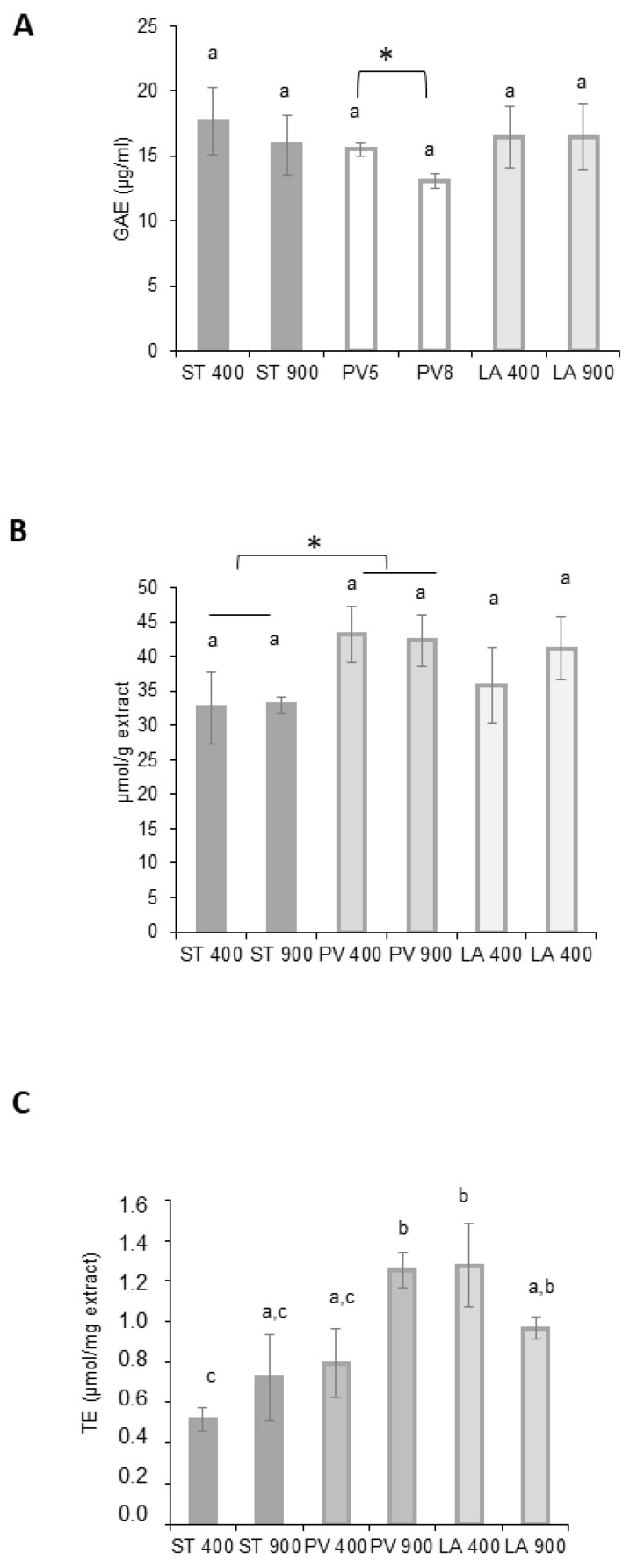
Antioxidant activity of Magic tomato fruit extracts. Fruit samples were collected from plants grown under ambient 400 ppm or elevated 900 ppm atmospheric CO_2_. (**A**) Total polyphenol content (Folin–Ciocalteu method); (**B**) 2,2-diphenyl-1-picrylhydrazyle (DPPH); (**C**) oxygen radical absorbance capacity (ORAC) assays. Means within each condition with the same letter are not significantly different according to one-way ANOVA followed by post hoc Tukey test or *t*-tests (*: significance: *p* < 0.05).

The total polyphenol contents of Magic tomato samples are represented in Figure 5A. The results indicate that the three tomato accessions had similar phenolic contents when exposed to ambient CO_2_. When considering eCO_2_ exposure, STUPICE and LA0147 TPC were not influenced by carbon dioxide enrichment. Conversely, the PLOVID variety was characterized by a significant decrease in TPC.

The antioxidant activity potential was further evaluated using two other methods: the DPPH and ORAC tests. The DPPH results are presented in Figure 5B. The free radical scavenging capacity was not affected by eCO_2_ in all of the tomato samples. Nonetheless, the PLOVID variety demonstrated a significantly higher antioxidant potential when compared to the STUPICE variety. The ORAC results are presented in Figure 5C. The eCO_2_ condition induced a significant increase in the antiradical activity of PLOVID accession, while it was not affected by eCO_2_ in both STUPICE and LA0147 tomato extracts.

### 2.5. Anti-Inflammatory—Immunomodulatory Bioactivity

#### 2.5.1. Nitric Oxide Production

**Figure 6:**
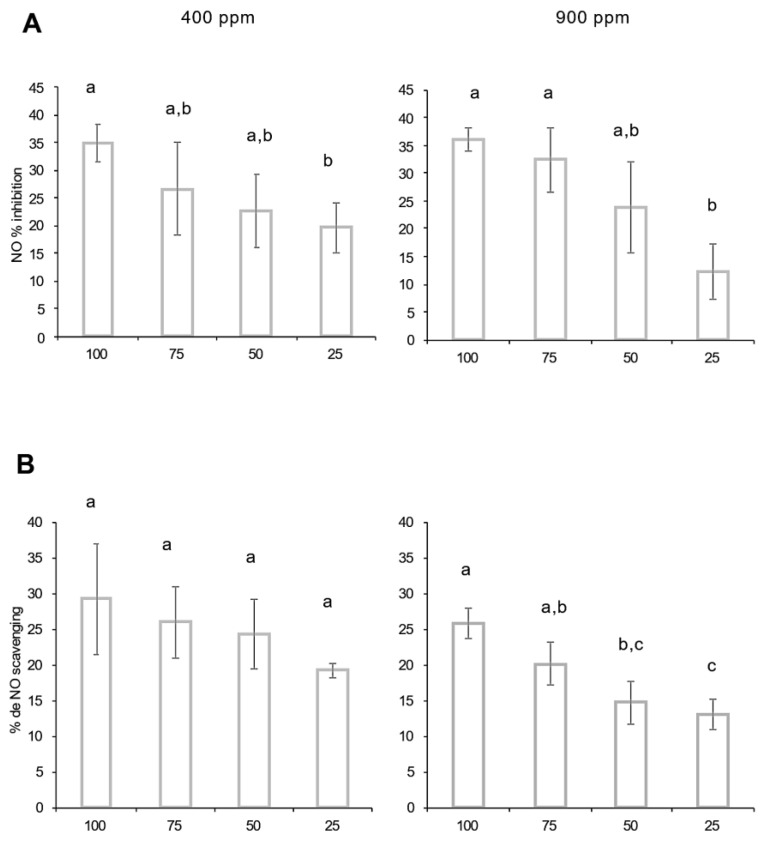
Effect of Stupice tomato fruit extracts on nitric oxide liberation and scavenging capacity. Fruit samples were collected from plants grown under ambient 400 ppm or elevated 900 ppm atmospheric CO_2_. (**A**) Fruit extract inhibition rates on nitric oxide production by J774 macrophages; (**B**) fruit extract nitric oxide scavenging capacity. Means within each condition with the same letter are not significantly different according to one-way ANOVA followed by post hoc Tukey test or *t*-tests (significance: *p* < 0.05).

The immunomodulating effects of Magic ethanolic extracts were assessed on the STUPICE accession. The results are presented in Figure 6A. STUPICE ethanolic extracts decreased nitric oxide (NO) production using stimulated macrophage cells. A similar concentration-dependent inhibition of NO production was observed in both high and ambient CO_2_ conditions. However, no significant difference was recorded between 400 ppm and 900 ppm exposures.

#### 2.5.2. Cytokines Interleukin-6 and TNF-α

**Figure 7:**
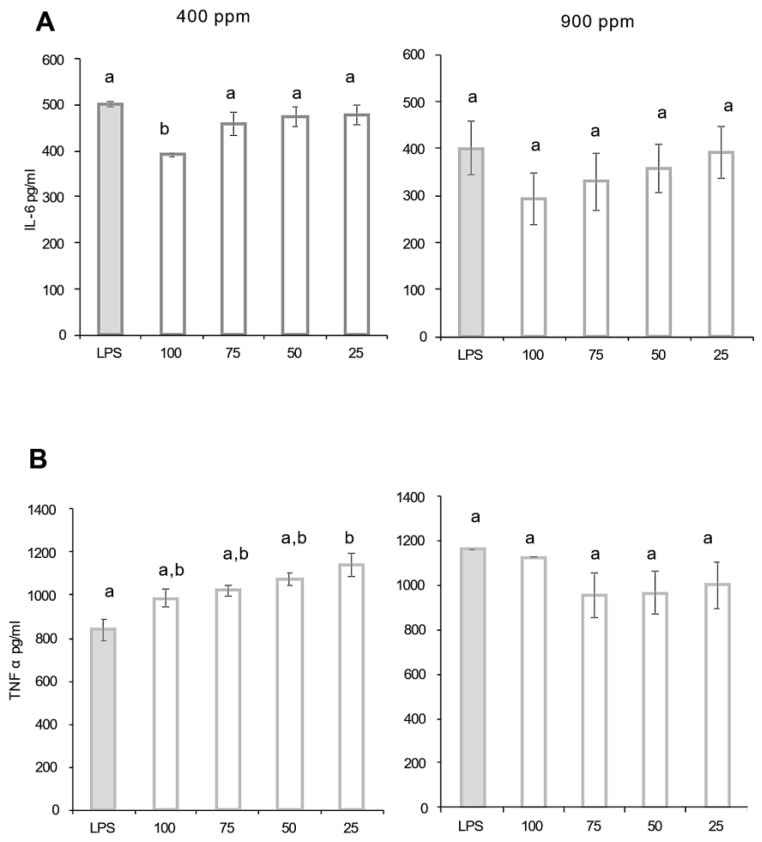
Anti-inflammatory activity of Stupice tomato fruit extracts. Fruit samples were collected from plants grown under ambient 400 ppm or elevated 900 ppm atmospheric CO_2_. (**A**) Interleukin 6 (IL-6); (**B**) tumor necrosis factor alpha (TNF-α) production by J774 macrophages stimulated with LPS (lipopolysaccharide) and interferon gamma (IFN-γ) were measured using ELISA technique. Means within each condition with the same letter are not significantly different according to one-way ANOVA followed by post hoc Tukey test or *t*-tests (significance: *p* < 0.05).

The results of STUPICE ethanolic extract anti-inflammatory activity measured using the ELISA sandwich method on Interleukin-6 (IL-6) and tumor necrosis factor alpha (TNF-α) are presented in Figure 7A,B. Regarding IL-6, tomatoes grown under ambient CO_2_ conditions significantly inhibited IL-6 production only at the highest concentration (100 µg/mL), while there was no inhibitory effect at lower concentrations. Under the eCO_2_ condition, STUPICE samples did not exhibit any repression effect on IL-6 production, whatever the concentration.

When considering TNF-α, macrophage cells treated with STUPICE extracts showed the same levels of TNF-α production than the control cells. Therefore, eCO_2_ treatment had no significant effect on the LPS/IFN-stimulated macrophage response.

## 3. Discussion

Our previous results on the MicroTom tomato model [23] confirmed a real alteration in tomato fruits’ composition and nutrition–health potential induced by elevated CO_2_ concentrations (eCO_2_: 900 ppm). The present study aimed to verify the impact of elevated atmospheric CO_2_ on other tomato accessions, i.e., Magic, which represent a large part of the genetic variability of all consumed tomato varieties with large fruits.

Tomato fruits and tomato products represent a good source of bioactive compounds. Minerals, vitamins C and E, β-carotene, lycopene, flavonoids, organic acids, and phenolics are some of the nutrients that support health functions and body balance [23,24].

Regarding tomato mineral content evolution as a function of CO_2_ exposure, the literature reports are scarce and indicate that it would influence plants’ mineral compositions, both directly and indirectly. Food crops’ mineral content alterations may therefore represent a potential health threat in the long run [1,23]. In this context, the recorded tendency indicates an irregular decrease or no modification as a function of the mineral observed. Our results for the Magic tomato suggest a trend of Na (sodium) content decrease in the three studied accessions. Minerals such as K, Cu, and Fe were not modified when Mn and Zn either decreased or were not modified. Finally, regarding Ca, it either decreased or strongly increased. When compared to our previous report [23], it appears that eCO_2_ exposure tends to have less impact on the mineral content of Magic tomato when compared to MicroTom. This observation is of great interest since Magic accessions are closer to large fruit consumed tomatoes. Considering that MicroTom is a small-fruit laboratory tomato model, it may suggest that marketed large-fruit tomatoes could be less impacted by eCO_2_ regarding their mineral content balance.

These variations of mineral content in response to eCO_2_, as a function of the cultivar, would be due to a modification in stomatal conductance, which in turn alters the transpiration-induced sap flow and thereby the uptake of mineral elements present in the soil [8,9]. Alternatively, it is also proposed that mineral content variations could be due to a dilution mechanism resulting from the positive effect of eCO_2_ on biomass [8]. It is not well established whether large-fruited plants have metabolic behavior that may result in a different response to the elevated atmospheric carbon dioxide. In the light of our results and of the literature, it is conceivable that not all cultivars respond as homogeneously as initially thought. Our differential results between small and large tomato fruits clearly suggest a significant variability in the effects of atmospheric CO_2_ levels on the phytochemistry and biological properties of tomato cultivars. In-depth multiparametric comparative investigations depending on cultivar, genetics, and growing conditions are required to understand the potential cultivar-dependent mechanisms involved in the consequences of rising atmospheric CO_2_.

In any case, modifications of mineral diet intake may have potential consequences for health. Sodium is a major electrolyte for cell homeostasis, the transmembrane electrochemical gradient, osmotic balance, neuron and cardiomyocyte functions, as well as many cellular and extracellular processes. Therefore, sodium balance disorders, and more particularly, hyponatremia, are related to numerous pathologies such as neuronal, cardiac, arterial blood pressure, and kidney, hepatic, and endocrine diseases [25,26,27]. Similarly, zinc, an intracellular and extracellular signaling messenger, is the second-most abundant mineral in the human body. It is a component of over 200 enzymes mainly involved in metabolism, the immune system, cognition, oxidative stress management (zinc superoxide dismutase), and cell proliferation. Zinc intake variations may alter the homeostasis of any of these numerous biological functions [28,29,30,31,32]. Finally, manganese, a homeostatic, tightly controlled mineral because of its potential toxicity, is involved in development, growth, oxidative stress management (manganese superoxide dismutase), and mitochondrial function. Manganese supply alterations may favor neurological disorders (Parkinson’s, Alzheimer’s, and Huntington’s disease) [32,33,34,35,36,37]. In consequence, variations in mineral intake associated with elevated CO_2_-induced vegetal composition may be linked to significant consequences for human health.

In addition to minerals, elevated CO_2_ was associated with a set of modifications in plant macronutrient composition. A high photosynthesis range resulting in carbohydrate accumulation was confirmed in several food crops [38]. The quantification of carbohydrate in Magic tomato accessions did not show any increase. This may be explained by downregulation processes in response to long-term exposition to eCO_2_ (4–5 months in our study). This so-called “acclimation phenomena” is considered a potential reason behind photosynthesis repression in a large range of vegetables [39]. Lipid accumulation is regulated by photosynthesis events. Two Magic tomato accessions, namely, STUPICE and LA0147, showed an increase in the total lipid content as a response to eCO_2_. Several pieces of research on multiple food crops have confirmed a high lipid content under eCO_2_ [40]. This effect was even more noticeable on microalgae species [41]. Some investigations related these results to a nutrient limitation mechanism [42]. Regarding proteins, several studies reported that plants grown under eCO_2_ presented a modified and low protein rate. Similarly, the total proteins of our three Magic accessions decreased significantly with eCO_2_ treatment, thereby affecting their nutritional value and potentially consumer physiological homeostasis [43]. It should be noted that with a decreased total protein, even though carbohydrate did not increase in our study, the carbon-to-nitrogen ratio increased anyway. Therefore, this general trend reported in the literature remains true for Magic tomato [38].

Considering micronutrients, tomato carotenoids and especially lycopene are considered key nutrients for antioxidant defenses. These pigments, which are present in Magic tomatoes, modulate antioxidant enzymatic reactions and protect against several diseases. Total carotenoids and lycopene content were significantly reduced in response to eCO_2_ in the three tomato samples, STUPICE, PLOVID, and LA0147. Our results are in agreement with other reports [44], demonstrating that lycopene accumulation also depends on CO_2_ levels. Furthermore, in a previous study, the tomato lycopene content was reported to be higher at 550 ppm and lower at 700 ppm in comparison with control conditions [45]. Our results obtained at 900 ppm suggest that increasing CO_2_ level exposure may further deteriorate tomato carotenoid compound content.

Based on Magic tomato chemical modifications induced by eCO_2_ exposure, biological properties were assessed to identify potential alterations. The literature reports suggest that the DPPH activity was improved by high CO_2_ concentrations_._ The antioxidant activity seems to be enhanced with CO_2_ in FRAP (Ferric Antioxidant Power) assays too [46]. Among other hypotheses, this might be explained by the upregulation of phenolic compounds, including flavonoids, such as in *Labisia pumila* leaves. ORAC scavenging properties and gluthation activity were also higher under eCO_2_ in strawberry fruits [47]. In tomato samples, elevated CO_2_ induced ROS inhibition, MDA (malondialdehyde) diminution, and antioxidant enzyme activation [48]. This could be explained by the fact that plants grown under eCO_2_ are characterized by a high photosynthesis rate and carbonic composite accumulation like phenolics and flavonoids. Conversely, other studies mentioned a reduction in phenolic, flavonoid, and FRAP activity under elevated CO_2_ [45,49]. Magic tomato demonstrated an anti-oxidant bioactivity, but in our case, eCO_2_ did not influence this capacity (except in the PLOVID accession). These observations, combined with the literature, suggest that the plant response to eCO_2_ may vary as a function of accessions and growth conditions, including the precise level of eCO_2_ and temperature [45,49].

Previous studies have demonstrated that tomato aqueous extracts promote an anti-inflammatory response in RAW macrophage cells by decreasing chemokine production (IL-6, TNF-α, …) and their regulation pathways [50]. It is well demonstrated that lycopene has antioxidant and anti-inflammatory properties. The inhibition of gene expression of several biomarkers is one of the proposed mechanisms [51]. Moreover, other mechanisms, such as the inhibition of NF-κB, might induce IL-6 and IL-8 downregulation, which may explain tomato potential bioactivity [52]. The anti-inflammatory properties of crops grown under high CO_2_ enrichment were improved in many species, like lemongrass [53]. For example, eCO_2_ significantly decreases COX-2 expression in broccoli [53]. In our case, the Magic tomato STUPICE demonstrated an anti-inflammatory effect characterized by NO scavenging capacity and inhibition of macrophage NO production. These properties were not influenced by eCO_2_. Regarding IL-6 and TNF-α productions, they were not influenced. These results may suggest that under eCO_2_ exposure, the type of accession and precise growth conditions at both plant nutrient, soil nature, temperature, and metabolic acclimation levels may be part of a complex and intricate network of influences leading to the specific response of the tomato plants.

## 4. Materials and Methods

### 4.1. Plant Materials and Sample Preparation

Seeds of tomato variety Solanum licopersicum Magic (STUPICE, PLOVID, and LA0147) were sterilized according to the method described by Appenroth [54,55] and germinated as described by Boufeldja et al., 2022 [23]. After germination, the plants were transferred to soil potting and kept in culture chambers under the following conditions: temperature of 22–23 °C and two different concentrations of CO_2_ (ambient CO_2_: 400 ppm and eCO_2_: 900 ppm). The plants were sprinkled with a fertilizer solution (N,P,K 6-13-18 with oligo-elements). The tomato fruits were harvested at the last stage of ripening (red color) then stored at −80 °C to be freeze-dried in CryoneXt lyophilizer (Thermofisher, France).

### 4.2. Mineral Content

Samples were collected from three independent plants (each accession is represented by 3 plants). Freeze-dried tomato samples were used for analysis. Around 10 mg of each sample was digested with 250 μL of 30% H_2_O_2_ as well as 750 μL of 65% nitric acid in 15 mL Digestion Cups (VWR, Radnor, PA, USA), and the tubes were degassed overnight. On the following day, the samples were incubated in the HotBlock (OnBoard, Meylan, France) for 8 h at 85 °C. After cooling, 4 mL of MilliQ water was added, and the samples were transferred to a 16 mm OD polypropylene tube (Agilent Technologies, Santa Clara, CA, USA). Mineral content of each sample was analyzed in technical triplicates using the 4100 MP-AES (microwave plasma atomic emission spectrometry, Agilent Technologies, Santa Clara, CA, USA) [23].

### 4.3. Micronutrients: Carotenoids and Lycopene Content

Extraction procedures and conditions for analysis were performed as follows: Freeze-dried Magic samples were weighed (50 mg) with 300 mg of sand in 15 mL tubes. The tomato samples were rehydrated with 1 mL of distilled water and homogenized. Then, 10mL of a solution of ethanol/hexane (4:3, *v*/*v*) containing 0.1% BHT was added. The mixture was homogenized using a Vortex and then Fast Prep^®^ 24 type agitation/grinding were applied at a speed of 6 m/s for 3 × 50 s. The hexane phase was collected in a 15 mL conical bottom falcon tube. The mixture was re-extracted twice in a row with 5 mL of hexane and Fast Prep 2 × 50 s. The hexane phases were then evaporated to dryness under nitrogen. Finally, the residue was re-dissolved in 500 μL of methyl tert-butyl ether (MTBE)/methanol (80:20 *v*/*v*) and 500 μL of dichloromethane and placed in an amber vial prior to HPLC analysis.

Carotenoid identification was performed on a reverse-phase HPLC DAD Agilent 1100 system (Agilent, Santa Clara, CA, USA) with a diode array detector. Carotenoids were separated using a C30 column (250 × 4.6 mm i.d., 5 μm) (YMC EUROP GmbH, Dinslaken, Germany) with a guard column, and the mobile phases were H_2_O as eluent A, methanol as eluent B, and MTBE as eluent C. Operation temperature was set at 25 °C. The flow rate was set at 1 mL/min, and the injection volume was 20 μL. A solvent gradient was programmed as follows: 0–2 min, isocratic 40% A—60% B (initial conditions); 2–5 min, 20% A—80% B; 5–10 min, 4% A—81% B—15% C; 10–60 min, 4% A—11% B—85% C; 60–70 min, isocratic 4% A—11% B—85% C; 70–71 min, 100% B; 71–72 min, with a return to the initial conditions for rebalancing. E-β-carotene and its isomer were detected at 450 nm, and *E*-lycopene and its isomers were detected at 470 nm. Isomers were identified according to their relative retention times, i.e., elution order, and the combined use of their spectral data. The identifications were based on previously published data obtained with the same mobile phase (water/methanol/MTBE) and the same detection wavelength range [23].

### 4.4. Macronutrients

#### 4.4.1. Sugars

Simple soluble sugars were analyzed by HPLC. Briefly, 500 mg of sample was rehydrated with 2 mL of distilled water and homogenized. Tomato samples were extracted three times with 10 mL ethanol (80%), then the mixture was heated at 70 °C for 10 min. After agitation for 20 min and centrifugation (4000× *g*, 5 min, 15 °C, Beckman Coulter, Brea, CA, USA), the supernatant was filtered through a 0.45 μm membrane before injection in UPLC. Samples were analyzed using a UPLC–1290 System Infinity II (Agilent, Santa Clara, CA, USA) equipped with a refractometer detector. A SHODEX SH1011 column 300 × 8 mm (Tokyo, Japan) was used with an isocratic system of water with H_2_SO_4_ (0.01%) and a flow rate of 0.7 mL/min. Temperature was set at 30 °C, injection volume at 10 μL, and spectrophotometric detection at 210 and 245 nm. External calibration was established for each standard sugar for concentrations from 0 to 10 g/L.

#### 4.4.2. Lipids

Lipid content was analyzed using the Folch method. A total of 500 mg of freeze-dried tomato samples were extracted three times with 15 mL of chloroform/methanol (2:1) solution. The mixture was agitated for 2 h and then centrifuged (6000 rpm, 10 min, 15 °C, Beckman Coulter). The supernatants were combined, then evaporated to dryness using a vacuum evaporation system (GeneVac EZ-2, SP Scientific, Warminster, PA, USA), and the tubes were weighed for the second time to determine the lipid content.

#### 4.4.3. Proteins

Protein content was calculated from nitrogen content assessed by the Kjeldahl method (Tecator Kjeltec) using a 6.25 conversion factor.

### 4.5. Antioxidant Bioactivity

Three different measurements were used to assess the antioxidant bioactivity of Magic tomato samples: (i) the total polyphenol content, (ii) the ORAC (oxygen radical absorbance capacity) assay, and (iii) the DPPH (2,2-Diphenyl-L-picrylhydrazyl) assay.

#### 4.5.1. Total Polyphenol Content

Total polyphenol assay was performed with Folin–Ciocalteu reagent, according to the method of Morel et al. [56]. Extracts of Magic and rosemary (*Rosmarinus Officinalis*) were prepared in DMSO at 4 mg/mL and then diluted in water to be tested at a concentration of 1 mg/mL. A calibration curve was generated on a concentration range from 1.56 to 75 μg/mL of gallic acid. In a 96-well plate, 50 μL of extract, 50 μL of gallic acid, and 50 μL of distilled water were distributed in triplicate. Then, 50 μL of 10% Folin–Ciocalteu reagent and 50 μL of sodium carbonate solution (1 M) were added. After 60 min in the dark, the absorbance was measured on a microplate reader (Molecular Devices, San Jose, CA, USA) at a wavelength of 650 nm. Results were expressed as milligrams of gallic acid equivalents (GAEs) per gram of Magic extract.

#### 4.5.2. ORAC (Oxygen Radical Absorbance Capacity) Assay

The ORAC assays were performed in 96-well opaque polypropylene plates as previously described [23]. Samples were solubilized in DMSO at a concentration of 1 mg/mL before being diluted to 25 μg/mL using phosphate buffer at pH 7.4. On the 96-well microplate, 20 μL of Trolox solutions at 0.6, 25, 12.5, 25, 50, and 75 μM as standard curve, or chlorogenic acid (0.01 mg/mL), or ethanolic extract of rosemary (12.5 μg/mL) as a positive control, or the extracts at a concentration of 25 μg/mL, were applied. Then, 100 μL of phosphate buffer and 100 μL of extemporaneously prepared fluorescein solution (0.1 μM in phosphate buffer) were added. The microplate was incubated at 37 °C for 10 min with shaking. The reaction was initiated with 50 μL of AAPH. Fluorescence was recorded at an excitation wavelength of 485 nm and an emission wavelength of 535 nm for 70 min using a Tristar LB 941 microplate reader. Final ORAC values were calculated using a regression equation between Trolox concentration and area under the curve of decreasing fluorescein. Data are expressed as μmoles of Trolox equivalents per gram of dry extract.

#### 4.5.3. DPPH (2,2-Diphenyl-l-picrylhydrazyl) Assay

Antioxidant activity was evaluated using the DPPH assay according to the method described previously [56]. Extracts were solubilized in DMSO (4 mg/mL) before being diluted in absolute ethanol to reach a concentration of 1 mg/mL. A standard curve of Trolox was performed (75, 50, 25, and 12.5 μM). Ethanol was used as blank, and ethanolic extracts of rosemary (0.2 mg/mL) and chlorogenic acid (0.01 mg/mL) were used as positive controls.

In a 96-well plate, 100 μL of positive control or extract was placed in each well. The test was performed in triplicate for each extract. A total of 75 μL of absolute ethanol and 25 μL of extemporaneously prepared DPPH solution (0.4 mg/mL) were introduced into each well. The plate was incubated for 30 min at room temperature and protected from light. The absorbance was read at 550 nm with a microplate reader (MDS Inc., Toronto, ON, Canada). Results were expressed as the mean plus or minus standard error to the mean of three independent experiments and were expressed as Trolox equivalents (TE μmoles per gram of dry extract).

### 4.6. Immunomodulatory Anti-Inflammatory Activity on Macrophage Cells Culture

Anti-inflammatory activity was assessed on the Stupice accession under both ambient CO_2_ and eCO_2_ conditions.

#### 4.6.1. Macrophage Culture

The macrophage cell line J774.A1 (ATCC and TIB67) was obtained from LGC Standards (Manchester, NH, USA). Cells were cultured in RPMI 1640 GlutaMAX^®^ medium supplemented with streptomycin (100 μg/mL) and penicillin (100 units/mL), 10% inactivated fetal calf serum (complete RPMI medium), and cells were incubated at 37 °C, 5% CO_2_, and 95% humidity.

#### 4.6.2. Cell Viability Assay

To test cytotoxicity, 6 × 10^5^ cells/well were seeded in a 96-well culture plate in complete RPMI medium and incubated at 37 °C with different concentrations of extracts (25, 50, 75, and 100 μg/mL) for 20 h. After incubation, 20 μL/well of (3-(4,5-dimethylthiazol-2-yl)-5-(3-carboxymethoxyphenyl)-2-(4-sulfophenyl)-2H-tetrazolium), MTS, mixed with an electron coupling reagent, PMS in HBSS, was added. The plate was incubated for an additional 4 h, and the absorbance at 490 nm was measured in a microplate reader (Molecular Devices, San Jose, CA, USA), as previously described [57].

#### 4.6.3. Dosage of NO (Nitric Oxide), IL-6 (Interleukin-6), and TNF-α (Tumor Necrosis Factor Alpha)

J774.A1 cells were seeded on a 24-well culture plate with complete RPMI medium. They were pretreated with various concentrations of Stupice extracts of 100, 75, 50, and 25 μg/mL for 4 h, stimulated with LPS (100 ng/mL) and interferon γ (10 ng/mL), and incubated for another 16–18 h at 37 °C. Supernatants were collected for nitrite determination or stored at −80 °C until use for TNFα and IL-6 dosages.

##### Determination of Nitrites (NO)

The presence of nitrite, a stable oxidized product of nitric oxide, was determined in the cell culture media as previously described [57]. Briefly, 100 μL of supernatant was combined with 100 μL of Griess reagent in a 96-well plate, and incubated for 10 min at room temperature. Nitrite concentration was determined by measuring absorbance at 550 nm and using a NaNO_2_ standard curve (from 1.56 to 100 μM). Results were expressed as a percentage of inhibition values.

##### Interleukin 6 (IL-6) Assay

IL-6 production by J774 cells was determined using the IL-6 ELISA kit (Mouse IL6 ELISA; Thermo Fisher Scientific, Vienna, Austria) after pretreatment with Stupice extracts at a determined concentration range (25, 50, 75, and 100 μg/mL) for 18 h. The cells were stimulated with 100 ng/mL LPS (*Escherichia coli*, 555B5) and 10 ng/mL mouse INF-γ for 4 h. IL-6 release in cell supernatants was tested according to the ELISA kit instructions. The results for IL-6 as well as for all other pro-inflammatory cytokines are expressed as a percentage of inhibition values.

##### Tumor Necrosis Factor Alpha (TNF-α) Assay

The TNF-α assay was performed according to the instructions contained in the ELISA kit (TNF alpha Mouse Uncoated ELISA kit; Thermo Fisher Scientific). After pretreatment with the different concentrations of Stupice extracts for 3 h, the cells were stimulated with LPS 100 ng/mL (*E. coli*, 555B5) and mouse INF-γ 10 ng/mL for 4 h. TNF-α release in cell supernatants was tested by sandwich enzyme-linked immunosorbent ELISA assay.

### 4.7. Statistical Analysis

All statistical analyses were performed using XLSTAT software version 2019.4.1 (Addinsoft, Paris, France). All data were reported as means ± standard deviation to the mean (SD) from three replicates of each experiment. One-way ANOVA followed by post hoc Tukey test or *t*-tests (significance: *, *p* < 0.05 and **, *p* < 0.01) were used.

## 5. Conclusions

In the present study, we investigated the impact of an elevated atmospheric carbon dioxide concentration (eCO_2_: 900 ppm) on the nutrition–health properties of tomato *Solanum lycopersicum* L. Magic accessions. Taken together, our results reveal two main points: First, Magic tomato demonstrated differential behavior when exposed to eCO_2_. Indeed, surprisingly, sucrose, fructose, and glucose were not increased by eCO_2_ as one would have expected based on classical C3 plant reports. Conversely, the protein content decreased, as commonly observed with C3 plants in the literature, with a still increased carbon-to-nitrogen ratio. Similarly, carotenoid and lycopene contents also decreased in response to elevated CO_2_. Second, when considering antioxidant properties, i.e., TPC, DPPH, and ORAC tests, the results did not show any influence of eCO_2_ exposure when compared to ambient CO_2_ exposure. In addition, regarding anti-inflammatory properties, if Magic STUPICE inhibited NO production, the eCO_2_ growing condition did not influence neither NO production, NO scavenging, nor IL-6 or TNF-α production. Therefore, our results suggest that elevated atmospheric CO_2_ appears to have a differential impact on C3 plants as a function of the genetic background. Many hypotheses may be proposed to try to explain these observations, among which, but not restricted to, plants’ nutrient availability, plants’ short-term adaptation, etc. In any case, this could mean that the eCO_2_ impact on tomato and more generally C3 plant nutrition health potential may not be influenced in an identical monolithic way, i.e., decreased protein content, increased carbohydrate content, and decreased biological activities. Plant responses may be modulated in a more complex and accession-dependent way when exposed to eCO_2_.

Therefore, further investigations are clearly needed to unravel the differential impacts of atmospheric eCO_2_ on the different C3 plant species as well as the genetic and physiologic determinants of their respective specific adaptative responses. This would provide valuable insight to this fundamental research question. It would contribute to optimize decision-making about the management of agro-food health systems for food crops in general and for tomatoes in particular. This is of significant importance for population health as consumers have access to a wide variety of accessions with potentially large variations in their nutrition–health qualities. Such explorations on the influence of eCO_2_ on various tomato accession phytochemistry and nutrition–health profile variations would help anticipate the putative final impact on the health of healthy and pathological populations. With climate change becoming more significant over the years, these issues should be anticipated to better face the human need for healthy food, contributing to the prevention of non-communicable metabolic diseases.

## Data Availability

Not applicable.

## Sample Availability

Samples of the compounds are available from the authors.
